# Unveiling the Significance of FGF8 Overexpression in Orchestrating the Progression of Ovarian Cancer

**DOI:** 10.3390/ijms241814239

**Published:** 2023-09-18

**Authors:** Kumari Binita Chandra, Vikrant Kumar, Swati Ranjan, Abhinav Saini, Anil Kumar Tomar, Jai Bhagwan Sharma, Sandeep R. Mathur, Savita Yadav

**Affiliations:** 1 Department of Biophysics, All India Institute of Medical Sciences, New Delhi 110029, India; binita.tiwary23@gmail.com (K.B.C.); vikrant.chikara@rediffmail.com (V.K.); swatiiranjan9818@gmail.com (S.R.); abhisan.bt@gmail.com (A.S.); aniltomar@aiims.edu (A.K.T.); 2 Department of Obstetrics and Gynecology, All India Institute of Medical Sciences, New Delhi 110029, India; jbsharma2000@gmail.com; 3 Department of Pathology, All India Institute of Medical Sciences, New Delhi 110029, India; mathuraiims@gmail.com

**Keywords:** cell invasion, cell migration, FGF8, gene silencing, ovarian cancer

## Abstract

The asymptomatic nature, high rate of disease recurrence, and resistance to platinum-based chemotherapy highlight the need to identify and characterize novel target molecules for ovarian cancer. Fibroblast growth factor 8 (FGF8) aids in the development and metastasis of ovarian cancer; however, its definite role is not clear. We employed ELISA and IHC to examine the expression of FGF8 in the saliva and tissue samples of epithelial ovarian cancer (EOC) patients and controls. Furthermore, various cell assays were conducted to determine how FGF8 silencing influences ovarian cancer cell survival, adhesion, migration, and invasion to learn more about the functions of FGF8. In saliva samples, from controls through low-grade to high-grade EOC, a stepped overexpression of FGF8 was observed. Similar expression trends were seen in tissue samples, both at protein and mRNA levels. FGF8 gene silencing in SKOV3 cells adversely affected various cell properties essential for cancer cell survival and metastasis. A substantial reduction was observed in the cell survival, cell adhesion to the extracellular matrix, migration, and adhesion properties of SKOV3 cells, suggesting that FGF8 plays a crucial role in the development of EOC. Conclusively, this study suggests a pro-metastatic function of FGF8 in EOC.

## 1. Introduction

Ovarian cancer is a multifactorial disease that may originate from the ovaries or nearby structures such as the fallopian tubes and the peritoneum. According to GLOBOCAN 2020, it ranks as the seventh most common cancer worldwide. The incidence rate of ovarian cancer and deaths caused by it will increase by 42% and 50%, respectively, globally by 2040 [1]. Approximately 85–90% of total ovarian cancer cases are epithelial ovarian cancer (EOC). The possibility of developing ovarian cancer is significantly increased by several risk factors. A family history of early-stage ovarian or breast cancer is a significant risk factor for the development of ovarian carcinoma [2]. Mutations in the tumor-suppressing genes BRCA1 and BRCA2 as well as the MMR gene are predominantly linked to the genetic risk of ovarian cancer and may increase that risk from 1.6% to 40%, 18%, and 10%, respectively [3]. As per studies, ovarian cancer risk increases with the number of ovulatory cycles a woman completes [4]. Debulking surgery combined with chemotherapy and radiation therapy constitutes the standard course of treatment for ovarian cancer. The current recommendations of the National Comprehensive Cancer Network (NCCN) for adjuvant chemotherapy include intravenous taxane/carboplatin and liposomal doxorubicin/carboplatin regimens [5]. Poly (ADP-ribose) polymerase (PARP) inhibitors are one recently developed treatment for ovarian cancer. These inhibitors prevent the regeneration process in cancer cells, preventing proliferation and the development of larger tumors [6]. The high death rate amongst EOC patients is mainly due to its asymptomatic nature, high rate of disease recurrence, and resistance to platinum-based chemotherapy [7]. Current treatment options, such as surgery, chemotherapy, and targeted therapies, have limitations in terms of efficacy and tolerability, highlighting the need to identify and characterize novel targets for the development of more effective and targeted therapies. Moreover, identifying novel targets can contribute to the early detection and prevention of ovarian cancer.

Fibroblast growth factor 8 (FGF8) is a 26 kDa polypeptide growth factor that possesses mitogenic and cell survival activities. It has been found to be involved in different biological processes, such as embryonic development, cell growth, morphogenesis, tissue repair, tumor growth, and invasion [8]. FGF8 is highly expressed during embryonic development and several malignancies such as breast, ovarian, and prostate cancer, whereas, in normal adult tissues, its expression is much more restricted. FGF8 interacts with FGF receptors (FGFRs) to regulate fundamental developmental pathways during embryo development and also controls events such as cell proliferation, differentiation, cell survival, angiogenesis, and wound healing in cancer cells [9,10,11]. Although FGF8 has a higher affinity for FGF receptors (FGFRs), FGFs also have a low affinity for heparin sulfate proteoglycans [12] and cysteine-rich FGF receptors [13]. Heparin sulfate proteoglycans control FGF8 signaling and its interaction with FGFR [14,15], resulting in the activation of various cell signaling cascades such as phospholipase C gamma, phosphatidylinositol-3 kinase, and MAPK pathways. Recently, overexpression of FGF8 was found to be associated with tumor progression in the clinical samples of colorectal cancer patients [16]. Additionally, FGF8’s expression level was found to be elevated in the tissue samples of oral squamous cell carcinoma and correlated with clinic-pathological factors such as age, alcohol consumption, and survival time [17]. Increased expression of FGF8 has also been reported in pancreatic ductal adenocarcinoma patients who have undergone neo-adjuvant therapy, suggesting that FGF8 is a prospective target for novel anticancer therapeutics [18]. However, no conclusive studies have highlighted a direct role of FGF8 in the progression of ovarian cancer. Therefore, expression analysis and functional characterization of FGF8 may offer significant evidence for comprehending the molecular mechanisms underlying the onset and progression of EOC.

## 2. Results

### 2.1. FGF8 Expression in Saliva Samples

**Figure 1:**
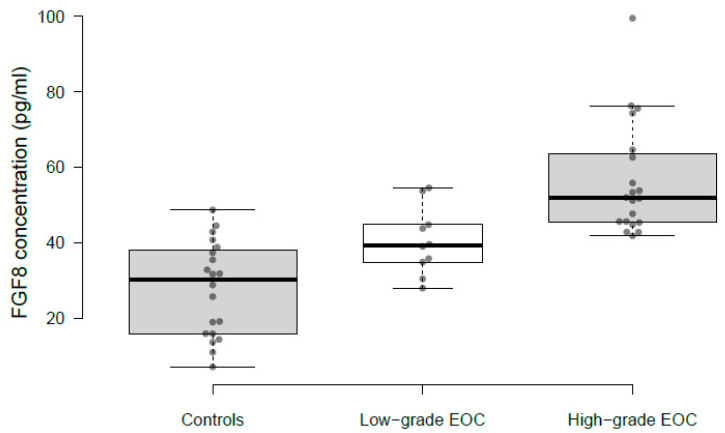
Box and whisker plot for FGF8 concentration in saliva samples of controls, low-, and high-grade epithelial ovarian cancer (EOC) as determined by ELISA. The center lines show the medians (30.27, 39.33, and 51.87); box limits indicate the 25th and 75th percentiles; whiskers extend 1.5 times the interquartile range from the 25th and 75th percentiles, a single outlier in high-grade EOC is represented by a dot; data points are plotted as open circles. n = 20, 10, 20 sample points. A sequential upward expression trend was observed from controls to low- to high-grade EOC with significant *p*-values; low-grade EOC vs. controls = 0.008 (two-tailed *t*-test); high-grade EOC vs. controls ≤ 0.0001 (Mann–Whitney test); and low- vs. high-grade EOC = 0.002 (Mann–Whitney test).

The Shapiro–Wilk test showed that data from control (W = 0.949, *p*-value = 0.358, coefficient of skewness = −0.03) and low-grade EOC (W = 0.946, *p*-value = 0.624, coefficient of skewness = 0.395) saliva samples were distributed normally, while data from high-grade EOC deviated from normality (W = 0.832, *p*-value = 0.003, coefficient of skewness = 1.511). Compared to controls, FGF8 was found to be overexpressed in both low- and high-grade EOC patients. In total, FGF8 was quantified in 30 EOC (10 low-grade EOC and 20 high-grade EOC) and 20 control samples (Figure 1). The average concentration of FGF8 was 27.79 ± 12.19 pg/mL (95% confidence interval (CI) = 21.93 to 33.64), 40.47 ± 8.46 pg/mL (95% CI = 34.09 to 46.85), and 56.38 ± 14.62 pg/mL (95% CI = 49.36 to 63.4) in controls, low-, and high-grade EOC, respectively. A sequential upward expression trend was observed from controls to low- to high-grade EOC with significant *p*-values, 0.008 (two-tailed *t*-test), in the case of low-grade EOC vs. controls, and <0.0001 (Mann–Whitney test) for high-grade EOC vs. controls. Moreover, FGF8 expression in low- and high-grade EOC also varied significantly, with an average fold change of 1.39 and a *p*-value = 0.002.

### 2.2. FGF8 Expression in Tissue Samples

**Figure 2:**
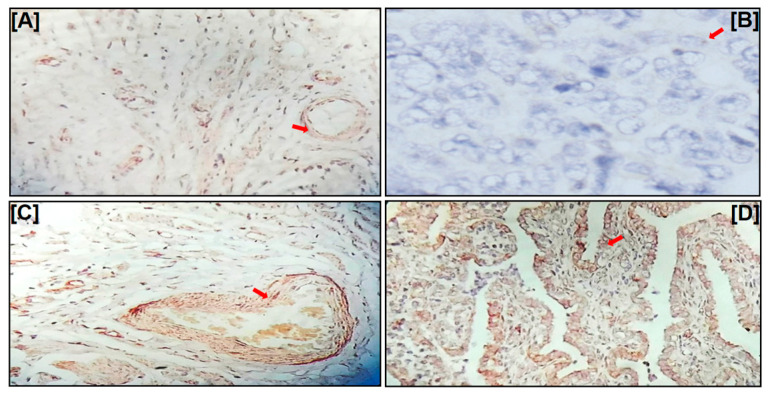
IHC staining of FGF8. (**A**) Fallopian tube tissue as positive control; (**B**) normal ovarian tissue with negative staining; (**C**) low-grade epithelial ovarian cancer (EOC) tissue with moderate staining; and (**D**) high-grade EOC tissue with strong staining. Arrows indicate the intensity of the stain (brown color). A total of 20 EOC tissue samples (10 each, low- and high-grade EOC) were examined.

FGF8 protein expression variation in tissue samples (n = 20, 10 each low- and high-grade EOC) was studied by IHC. The staining intensity of the cytoplasmic expression was scored 0 to 3 (0 = negative; 1 = weak; 2 = moderate; 3 = strong), while the percentage of positively stained cells was scored into four categories, 1 (0–25%), 2 (26–50%), 3 (51–75%), and 4 (76–100%). The immuno-reactive score (IRS) was used to evaluate the level of staining of the protein, simply calculated by multiplying the score of staining intensity and the percentage of positive cells. Staining intensity was qualitatively scored based on pathological guidelines in all tissue samples representing normal ovary, low-, and high-grade EOC. FGF8 staining was negative on normal ovary tissue sections, but it was abundantly present in the low-grade EOC, and its expression increased significantly with the stage advancement. Differential expression of FGF8 was detected in 80% of EOC tissue samples, and FGF8 expression increased with the progression of EOC (Figure 2).

### 2.3. FGF8 mRNA Expression by Quantitative PCR

**Figure 3:**
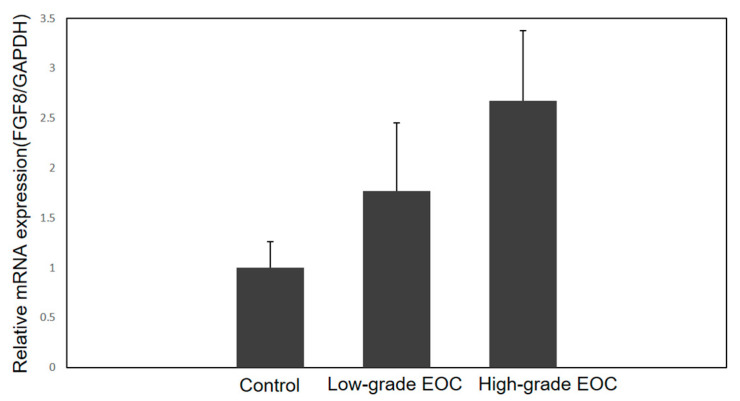
Relative mRNA expression of FGF8/GAPDH in tissue samples of controls, low-, and high-grade epithelial ovarian cancer (EOC) as determined by RT-PCR. Standard errors are also shown. Number of samples, n = 25 (8 controls, 5 low-grade EOC, and 12 high-grade EOC). 95% CI values: controls = 0.433 to 1.328; low-grade EOC = 0.213 to 2.903; high-grade EOC = 1.237 to 3.477. Comparative analysis: control vs. low-grade EOC: fold change = 1.8, *p*-value = 0.156; control vs. high-grade EOC: fold change = 2.7, *p*-value = 0.005.

Similar to saliva samples, the Shapiro–Wilk test showed that data from control (W = 0.931, *p*-value = 0.521, coefficient of skewness = 0.324) and low-grade EOC (W = 0.949, *p*-value = 0.727, coefficient of skewness = 0.96) tissue samples were distributed normally, while data from high-grade EOC tissue samples deviated from normality (W = 0.821, *p*-value = 0.018, coefficient of skewness = 1.778). Sixteen EOC (5 low- and 11 high-grade) and eight control tissue samples were processed for total RNA extraction and subsequent FGF8 mRNA quantitation by RT-PCR. Relative abundance and fold change analysis showed that compared to controls, FGF8 mRNA expression was 1.8- (*p*-value= 0.156, *t*-test) and 2.7 (*p*-value = 0.005, Mann–Whitney test)-fold higher in low- and high-grade EOC, respectively (Figure 3).

### 2.4. FGF8 Gene Silencing in SKOV3 Cells

**Figure 4:**
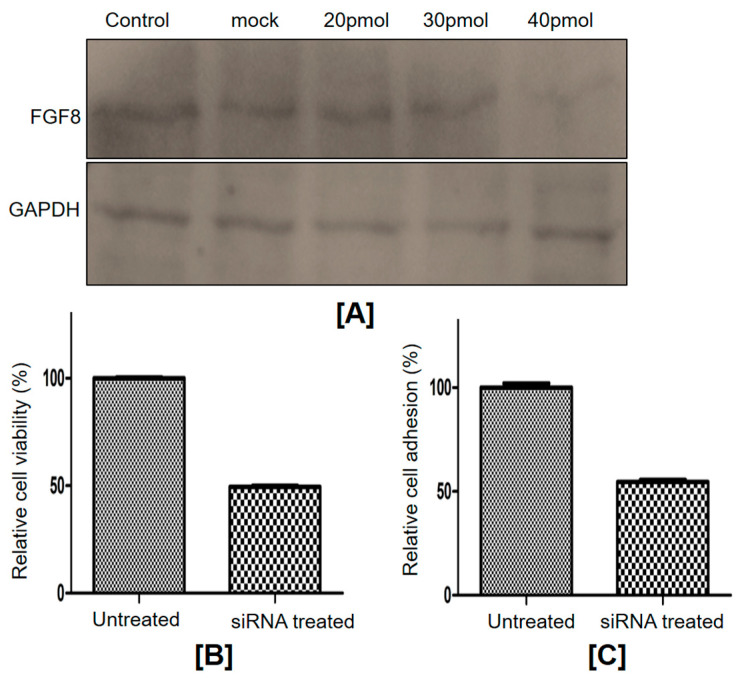
Effect of FGF8 knockdown on cell viability and adhesion of SKOV3 cells. (**A**) Western blot of FGF8 after siRNA transfection in SKOV3 cells displayed a significant reduction in FGF8 expression at a 40 pmol concentration of siRNA. GAPDH was used as an internal control; (**B**) MTT assay showed a significant reduction in cell viability of SKOV3 cells transfected with FGF8 siRNA compared to untreated cells, *p*-value = 0.0013; (**C**) adhesion analysis of SKOV3 cells. The result showed a significant reduction in the percentage of adherent cells in the FGF8 knockdown cells, *p*-value = 0.0001.

FGF8 gene silencing in SKOV3 cells was monitored by Western blotting. The results showed the least FGF8 expression with siRNA at a concentration of 40 pmol (Figure 4A). This highlights that a 40 pmol concentration of FGF8-specific siRNA was the most efficient and could significantly knock down FGF8 in SKOV3 cells.

### 2.5. Effect of FGF8 Silencing on Various Properties of SKOV3 Cells

**Figure 5:**
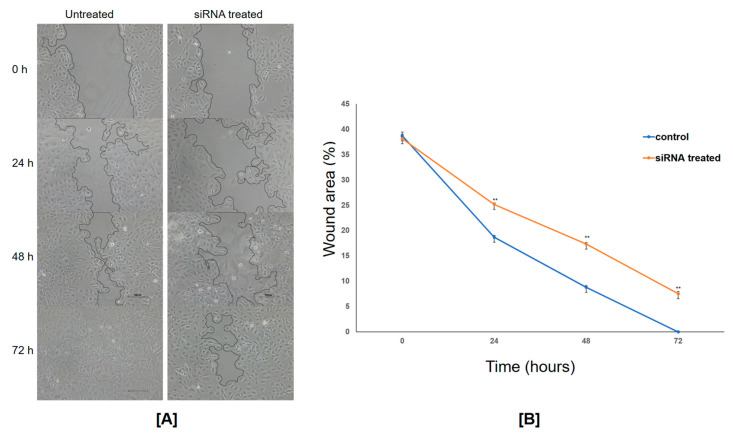
Wound healing assay. (**A**) Time-lapse images showing the progress of cell migration and wound closure in FGF8 siRNA-treated and -untreated cells. A scratch was made on a confluent cell monolayer, and images were captured at different time points to monitor the migration of cells into the wounded area. (**B**) Quantification of wound area. Line graph depicting the quantification of wound area in FGF8 siRNA-treated and -untreated cells. The width of the wound was measured at different time points. Data are presented as mean ± standard deviation; ** *p*-value threshold < 0.05. The wound area in the untreated cell monolayer was reduced significantly compared to FGF8 siRNA-transfected cells.

**Figure 6:**
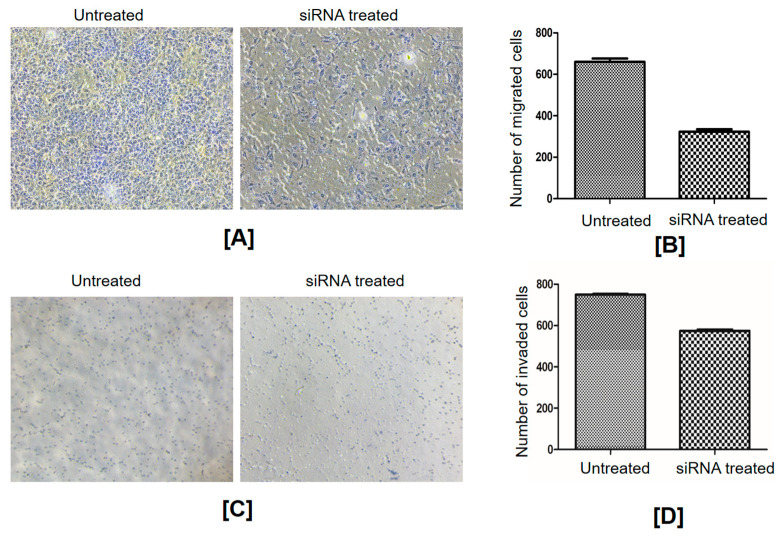
Cell migration and invasion. (**A**) Representative images of crystal-violet-stained migratory cells. FGF8 siRNA-treated and -untreated cells were seeded in the upper chamber and allowed to migrate toward a chemoattractant placed in the lower chamber. The migrated cells were stained and visualized under a microscope. (**B**) Quantification of cell migration. Bar graph showing the quantification of migrated cells in FGF8 siRNA-treated and -untreated groups. The number of migrated cells was counted and expressed as a percentage of the untreated group. Data are presented as mean ± standard deviation, *p*-value = 0.0028. (**C**) Microscopic images demonstrating the invasion of cells through a Matrigel-coated transwell membrane. Cells were seeded in the upper chamber and allowed to invade through the matrix toward a chemoattractant placed in the lower chamber. Invaded cells were stained and visualized under a microscope. (**D**) Quantification of cell invasion. Bar graph illustrating the quantification of invaded cells in FGF8 siRNA-treated and -untreated groups. Data are presented as mean ± standard deviation, *p*-value = 0.0017. Cell migration and invasion characteristics were significantly reduced in FGF8 knockdown SKOV3 cells.

A series of experiments were carried out to assess the effect of FGF8 gene silencing on SKOV3 cells. FGF8-expressing SKOV3 cells are referred to as control cells, whereas cells transfected with 40 pmol FGF8 siRNA are referred to as FGF8-silenced cells. First, an MTT assay was used to determine the effect of FGF8 gene silencing on the viability of SKOV3 cells. There was a significant difference in cell viability between the control cells and FGF8-silenced cells (Figure 4B). Compared to control cells, the viability of FGF8-silenced cells was reduced by ~50% (*p*-value = 0.0013). The effect of FGF8 silencing on the ability of ovarian cancer cells to adhere to the extracellular matrix (ECM) was investigated by adhesion assay, and it was observed that the adhesiveness of FGF8-silenced cells reduced by approximately 45% (*p*-value = 0.0001) (Figure 4C). Cell migration is an important property of cancer cells in the development of malignancy. As observed by the closure of the wound at different time points of incubation (24 h, 48 h, and 72 h), the rate of cell migration in FGF8-silenced cells was considerably lower than that in control cells (17.66%, 22.64%, and 19.66% at 24 h, 48 h, and 72 h, respectively; *p*-value threshold < 0.05) (Figure 5). These findings show that FGF8 knockdown slowed down the migration of the ovarian cancer cells, suggesting a conceivable involvement of FGF8 in ovarian cancer progression and metastasis. Furthermore, a migration assay was performed to better understand the correlation between the expression of FGF8 and the ability of ovarian cancer cells to migrate toward a chemoattractant. FGF8-silenced cells, in comparison to control cells, displayed about a 55% reduction in their ability to pass through the upper chamber membrane (*p*-value = 0.0028) (Figure 6A,B). In addition, FGF8 silencing altered the ability of SKOV3 cells to invade the ECM. After 48 h of incubation, cells could be seen invading through Matrigel 18; however, the invasiveness was ~30% lower (*p*-value = 0.0017) in FGF8-silenced cells than that in control cells (Figure 6C,D).

## 3. Discussion

Despite substantial advances in the fields of diagnostic, therapeutic, and surgical procedures, the prognosis of patients with advanced or recurrent states of EOC is discouraging. Current treatment options such as surgery, chemotherapy, and targeted therapies have limitations in terms of efficacy and tolerability. Identifying novel targets in ovarian cancer can lead to the development of new therapies. By targeting different molecular markers and associated pathways, it may be possible to bypass or reverse drug resistance, thus increasing the chances of successful treatment [7,19,20]. The human fibroblast growth factor (FGF) family is comprised of 22 different proteins regulating a variety of physiological functions. The majority of them are secreted proteins that function as growth factors by activating corresponding receptors [21]. The FGF8 subfamily is particularly intriguing as members of this family regulate embryonic development processes and aid in tissue repair in adults [22]. When first discovered, FGF8 was known as an androgen-induced growth factor released by a breast cancer cell line [23]. FGF8 is essential for embryonic development as it governs gastrulation and other early phases of development, and suppressing it in the germline causes embryonic lethality [24]. FGF8 has been shown to induce morphological changes, promote anchorage-independent cell proliferation in vitro, and promote tumor growth in vivo in breast and prostate cancer cells [25,26]. In adults, FGF8 is expressed at a low level in the kidney, breast, prostate, testis, and ovary. However, FGF8 expression alterations and its role in ovarian cancer remain unknown.

In this context, we employed ELISA and IHC to investigate the expression of FGF8 in the saliva and tissue samples, respectively, of EOC and controls to comprehend the connection between FGF8 expression and the development of EOC. To learn more about the functions of FGF8, it was knocked down using siRNA, and several cell assays were performed to see how FGF8 silencing affected ovarian cancer cell survival, adhesion, migration, and invasion. A sequential overexpression of FGF8 was observed in undiluted saliva samples, from controls to low-grade to high-grade EOC. During pathogenesis, overexpression of any protein can be cleaved from the cancer cells’ surface and shed directly into saliva. Salivary proteome shares one-fourth of plasma proteome and molecules from blood may reach into the saliva through various mechanisms such as ultrafiltration and trans-diffusion without any direct contact between them [27]. The exact mechanism related to the presence of FGF8 in the saliva is not fully understood, but it might be attributed to the passive diffusion of FGF8 from plasma into the saliva. Similar expression trends were observed in the tissue samples. Expression of FGF8 also changes at the mRNA level in ovarian and testicular cancers of different histological types, and its expression correlates with the tumor stage, and mRNA copy number variations generally result in the abnormal expression of a protein [8,28]. Therefore, we quantified FGF8 mRNA in tissue samples of EOC patients and controls using RT-PCR. A significantly higher expression of FGF8 mRNA was observed in advanced-stage EOC than in early-stage EOC, which endorses its association with cancer progression. Conclusively, a comparison of FGF8 expression in low- and high-grade EOC and control samples adds to the evidence that FGF8 expression correlates with tumor grade.

The mounting data suggest that FGF8 promotes carcinogenesis, progression, and metastasis in various cancers including ovarian cancer [16]. Its precise function in this regard and the underlying mechanisms, however, are largely unknown. To understand how FGF8 contributes to the development of EOC and to shed light on plausible molecular mechanisms, we knocked down FGF8 and studied various properties of cancer cells. FGF8 silencing showed a substantial growth inhibition effect against SKOV3 cells, highlighting that FGF8 facilitates ovarian cancer cells’ ability to survive for a longer period. Metastatic growth is controlled by the ECM and cell adhesion signaling [29]. The capacity of SKOV3 cells to attach to the ECM was disrupted when FGF8 was silenced, indicating that FGF8 plays a crucial role in ECM adherence. Based on these observations, it can be concluded that FGF8 promotes ovarian cancer progression and metastasis by facilitating ECM and cell adhesion signaling. For the current ovarian cancer treatment, metastasis remains a clinical challenge, and the determining element in metastasis is the ability of cancer cells to migrate to distant sites [30]. Cell migration and invasion are fundamental biological processes that play critical roles in numerous physiological and pathological conditions. These processes are involved in embryonic development, wound healing, tissue regeneration, and cancer metastasis [31]. Cell migration involves the movement of cells from one location to another. This process is essential for various physiological functions, such as immune surveillance, tissue morphogenesis, and wound healing. Cell invasion, on the other hand, involves the movement of cells through barriers, such as the ECM and basement membranes. Invasion is a crucial step in cancer metastasis, as cancer cells need to break through barriers to spread to distant sites in the body. The process of invasion is similar to that of migration but involves additional steps, such as the degradation of ECM components by proteases and the secretion of factors that promote angiogenesis and tissue remodeling [32,33]. ECM remodeling promotes the invasion and migration of cancer cells [34]. Thus, we investigated cell migration and invasion in FGF8-silenced SKOV3 cells and observed a significant reduction in these two cell properties, suggesting that FGF8 also helps ovarian cancer cells in migration and invasion.

## 4. Materials and Methods

All the reagents used in this work were of analytical grade and were purchased from Sigma-Aldrich, USA, unless stated otherwise.

### 4.1. Sample Collection

EOC patients and healthy controls were recruited from the Department of Obstetrics and Gynecology, All India Institute of Medical Sciences, New Delhi, after obtaining ethical clearance from the Institutional Ethics Committee (IEC/NP-162/2013). For this study, saliva and tissue samples were collected from pre-operated initial- and advanced-stage EOC patients and healthy controls. After informed consent was signed by the participants, unstimulated saliva samples were collected in the morning (between 9 am and 12 pm) from the participants either in the outdoor patient department (OPD) or in the patient ward by spitting directly in the sterile 50 mL vials. Soon after collection, samples were subjected to centrifuge at 12,000 rpm for 10 min at 4 °C, and the supernatant was collected and stored at −80 °C till further use. Saliva donors having any co-morbid condition, HIV infection, autoimmune disease, or habit of tobacco consumption were excluded from this study. Patients coming for the treatment of gynecological benign diseases and randomly selected age-matched healthy donors who consented to participate in the study were included as controls for saliva collection.

Tissue samples were collected from histo-pathologically confirmed initial- and advanced-stage EOC patients at the time of hysterectomy after the completion of a standard questionnaire and written informed consent. EOC patients receiving treatment in any form of chemotherapy or radiation were excluded from the study. The control tissue samples were collected at the time of salpingo-oophorectomy of the patients of gynecological benign diseases and were immediately preserved in RNA, later followed by storage at −80 °C till further use. Details of study participants are provided in Table 1.

### 4.2. Quantification of FGF8 in Saliva Samples

FGF8 expression levels in the saliva samples of EOC patients and healthy controls were measured by a commercially available ELISA kit (Biocodon, Mission, KS, USA) following the manufacturer’s protocol. Undiluted saliva samples were used and loaded in duplicate. FGF8 concentration in saliva samples is presented as mean ± standard deviation. To show ELISA results graphically, a box and whisker plot was constructed using BoxPlotR [35].

### 4.3. Extraction of Tissue Proteins

EOC and healthy control tissue samples were taken out from RNA later, sliced into small pieces, and washed with chilled 80% acetonitrile by centrifugation (3000 rcf, 10 min, 4 °C) to remove RNA later. After discarding the supernatant, the pellet was washed three times with phosphate buffer saline (PBS, pH 7.4), and suspended in an equal volume of RIPA buffer containing a protease inhibitor cocktail for 45 min at room temperature. Samples were then sonicated for 5 cycles of 3 s ON and 30 s OFF at 50 Hz, followed by centrifugation at 12,000 rcf for 15 min at 4 °C. The supernatant was then transferred to fresh tubes and subjected to protein precipitation in chilled acetone (1:4) at −20 °C for 6 h. Samples were then centrifuged at 12,000 rcf for 15 min at 4 °C, the supernatant was discarded, and the protein pellet was dissolved in 50 mM Tris buffer (pH 7.8). Protein concentration in each sample was estimated by Bradford assay as per the standard protocol.

### 4.4. Quantitative Real-Time PCR (RT-PCR)

Total RNA from EOC tissue samples was extracted using an RNeasy mini kit (Qiagen, Hilden, Germany) according to the manufacturer’s protocol, followed by treatment with DNase I to remove DNA traces. The quantity and quality of the total RNA were determined spectrometrically using a Nano-Drop 1000™ (Thermo Fischer Scientific, Waltham, MA, USA). Contamination was checked by the absorbance ratio A260/A280 for proteins and A260/A230 for phenol, polysaccharide, and/or chaotropic salts. Visualization of the 23S/16S banding pattern was used to assess the integrity of the total RNA.

One microgram of total RNA was used to prepare cDNA using a Script cDNA synthesis kit (Bio-Rad, Hercules, CA, USA) according to the manufacturer’s instructions. Real-time PCR was performed in a 10 μL reaction volume, containing SsoFast EvaGreen supermix qPCR (Bio-Rad, USA), 1 μL cDNA, and 1000 ng/μL final concentration of each primer. Primers used in RT-PCR experiments are described in Table 2. The qPCR run was performed on a CFX 96 (Bio-Rad, USA) with the following steps—(i) 95 °C for 30 s, (ii) 39 cycles of 95 °C for 5 s, (iii) Tm for 15 s, and (iv) 72 °C for 15 s. Relative fold change in each target mRNA transcript was calculated using the 2ΔCt method, ΔCt = Ct (housekeeping gene)-Ct (target gene). All experiments were performed in duplicate.

### 4.5. Immunohistochemistry (IHC)

Formalin-fixed paraffin-embedded tissues were collected from the Department of Pathology, AIIMS, New Delhi, and dewaxed at 65 °C for 30 min followed by three consecutive washes with xylene for 5 min. Rehydration of tissues was performed by washing the slides for 5 min sequentially with each 100%, 95%, 80% ethanol, and finally with distilled water. The slides were then heated to 95 °C for 30 min in 10 mmol/L sodium citrate (pH 6.0) for antigen retrieval, followed by treatment with 4% hydrogen peroxide for 30 min to block the endogenous peroxidase activity. After blocking the slides with the universal blocking serum, the sections were first incubated overnight with anti-FGF8 antibody at 4 °C, then with a biotin-labeled secondary antibody for 30 min, and finally with streptavidin–peroxidase (Zhongshan Biotech, Zhongshan, China). The slides were developed by treating with 3, 39-diamino-benzidine substrate (Zhongshan Biotech, China) and with hematoxylin to counterstain. Negative controls were developed by omitting the primary antibody incubation step.

### 4.6. Cell Culture and Transfection

Human ovarian cancer cells, SKOV3, were obtained from the National Centre for Cell Science, Pune, India, and cultured in modified McCoy’s 5A (Invitrogen Life Technologies, Carlsbad, CA, USA) containing 10% FBS and 0.3% penicillin/streptomycin. The cells were maintained at 37 °C in a 5% CO_2_ atmosphere. Antihuman FGF8 siRNA (Thermo Fischer Scientific, USA; product ID-HSS142008) was transfected into cells using Lipofectamine 2000 (Invitrogen, Waltham, MA, USA). Cells were collected 72 h post-transfection, and Western blotting was used to monitor FGF8 gene silencing.

### 4.7. Cell Viability Assay

MTT assay was used to determine cell viability after FGF8 gene silencing. For this, a 96-well plate with approximately 2 × 10^5^ cells was seeded, transfected, and then incubated at 37 °C in 5% CO_2_. After 72 h, the MTT reagent was added. The color development was measured at 570 nm after dissolving the MTT formazan crystals in 10% SDS.

### 4.8. Adhesion Assay

After 72 h, FGF8 siRNA transfected and control cells were seeded at a density of 2 × 10^4^ in (Corning Inc., New York, NY, USA) coated 96-well plates and allowed to adhere for 1 h. Non-adherent cells were washed away and adherent cells were stained with crystal violet for 10 min. After staining, crystal violet was dissolved in 10% acetic acid and measured at 590 nm.

### 4.9. Wound Healing Assay

After 24 h of growth, the cells were seeded into 6-well plates at a density of about 95% confluence. A 200 µL pipette tip was used to lightly scratch the monolayer across the well. The well was washed with PBS after scratching to remove the detached cells. An inverted microscope was used to monitor wound closure every 24 h after the scratch.

### 4.10. Cell Migration and Invasion Assay

For migration assay, FGF8-transfected and control cells were resuspended in serum-free media and seeded in the upper chamber of Transwell inserts (Corning Inc., USA) and allowed to migrate to a chemoattractant placed in the lower chamber. Migrated cells were stained with crystal violet and counted under a microscope. For invasion assay, the above-mentioned protocol was followed except that the upper chamber of Transwell inserts was coated with Matrigel 18 before the seeding of cells.

### 4.11. Statistical Analysis

The statistical analyses were performed in Excel and MedCalc statistical software package for biomedical research. Before analysis, the Shapiro–Wilk test was performed to check the normality of data distribution. A Student *t*-test was applied for comparing two data sets, while the Mann–Whitney test was performed in case of deviation from normality.

## 5. Conclusions

According to this study, FGF8 expression increases from control to low- to high-grade EOC samples, demonstrating a positive connection between tumor grade and FGF8 expression. Silencing the FGF8 gene in SKOV3 cells has a negative impact on the pro-metastatic cell characteristics, indicating that FGF8 is essential for cell survival and may help with the ECM and cell adhesion signaling, and migration. Conclusively, this research points to a pro-metastatic role of FGF8 in the development and progression of EOC.

## Figures and Tables

**Table 1:** Sample details.

Particulars	Saliva Samples			Tissue Samples		
Ethnicity	Indian			Indian		
Smoking History	No			No		
	Controls	Low-Grade EOC	High-Grade EOC	Controls	Low-Grade EOC	High-Grade EOC
Number of samples	20	10	20	8	5	11
Age (years) Mean ± SD	38.6 ± 7.0	42.6 ± 16.5	46.8 ± 10.2	47.0 ± 2.8	50.2 ± 14.4	44.9 ± 9.6

EOC: epithelial ovarian cancer; SD: standard deviation.

**Table 2:** Primer pairs used for FGF8 mRNA quantitation using RT-PCR.

Gene	Primers	T_m_ (°C)
*FGF8*	Forward: 5′TAGGGCACCCAAAACTCAAG3′	50.7
*GAPDH*	Reverse: 5′AACAGCAAAAACCCAACAGC3′	53.7

## Data Availability

Not applicable.
